# Factors influencing specialty choice and the effect of recall bias on findings from Irish medical graduates: a cross-sectional, longitudinal study

**DOI:** 10.1186/s12909-020-02405-w

**Published:** 2020-12-02

**Authors:** Frances M. Cronin, Nicholas Clarke, Louise Hendrick, Ronan Conroy, Ruairi Brugha

**Affiliations:** 1 Royal College of Surgeons in Ireland, Dublin 2, Ireland; 2 School of Psychology, Dublin City University, Glasnevin, Dublin 9, Ireland; 3 Dr Steevens’ Hospital, Health Service Executive, Dublin 8, Ireland

**Keywords:** Specialty choice, Medical, Doctor, Recall bias, Medical graduates, Medical training

## Abstract

**Background:**

Despite being a vital part of medical workforce planning and development, how medical students and graduates choose their career specialty is still not well understood. This study aimed to identify the factors medical graduates consider important influences in their choice of specialty after their first year of practice, and to test the validity of relying on respondent recall to measure changes in specialty choice.

**Methods:**

The baseline survey was administered online to all final year students in Ireland’s six medical schools. Those who consented to follow-up (*n* = 483) were surveyed 18 months later (June 2018), during the final month of first year of practice.

**Results:**

The baseline survey had a 67% (*n* = 483) response rate. At the follow-up survey, (*n* = 232, 48% response rate) the top specialty choices were: Medicine, *n* = 54 (26%); Surgery, *n* = 34 (16%); General Practice, *n* = 28 (13%); Anaesthesia, *n* = 16 (8%) and Paediatrics, *n* = 14 (7%). Of the 49 respondents (28%) reporting a change of specialty since baseline, 13 (27%) selected the same specialty in both surveys; of the 121 (69%) reporting no change, 22 (18%) selected a different specialty at follow-up.

Over 90% of respondents rated as ‘important or ‘very important’: ‘Own aptitude’, ‘Work-life balance’ and ‘What I really want to do’. Over 75% rated as ‘not at all’, or ‘not very important’ ‘Current financial debt’ and ‘Inclinations before medical school’.

When adjusted for sex and age, compared with Medicine, General Practice rated as more important: continuity of patient care (RRR 3.20 CI(1.59–6.41), *p* = 0.001); working hours/conditions (RRR 4.61 CI(1.03–20.60), *p* = 0.045) and a career that fit their domestic circumstances (RRR 3.19 CI(1.27–8.02), *p* = 0.014). Those choosing Surgery rated as less important: patient contact (RRR 0.56 CI(0.33–0.95), *p* = 0.033) and working hours/conditions (RRR 0.55 CI(0.31–0.96), *p* = 0.035).

**Conclusions:**

The different demographic and motivational profiles by specialty choice are consistent with other studies suggesting a distinct profile for doctors intending to enter General Practice. In addition, our results suggest longitudinal study designs guard against recall bias and so provide more robust medical workforce models to inform and direct recruitment drives and interventions in future medical workforce planning.

## Background

Today, there is a widely acknowledged crisis in medical doctor recruitment and retention, both in Ireland [1–4] and internationally [5–7]. In addition, Ireland is struggling to address an aging General Practice (GP) workforce and increasing losses through emigration, retirement and disenchantment [4, 8, 9]. The continued loss of domestically-trained doctors [1–3, 10] is being compounded by a shortage of EU-trained doctors and is resulting in Ireland increasingly relying on the recruitment of overseas-trained doctors, rising from 13% in 2000 [11] to 42% in 2017 [12]. This is in direct contravention of the WHO guidelines on international recruitment of medical personnel [12] to which Ireland is a signatory.

Despite being a vital part of medical workforce planning and development, how medical students and graduates choose their career specialty is still not well understood [13–17]. Numerous influencing factors have been proposed, including personal and socio-economic factors, such as sex and parental education [18, 19]; year of graduation and specialty characteristics [20]; specific job-related attributes, including a supportive culture and working conditions [21]; continuity of patient care [22]; costs associated with postgraduate training, particularly for surgery [23]; and original career choice on entry to study [24].

A review of 57 studies of career decision-making by medical students identified five broad categories of influence: (i) medical school characteristics (e.g. curriculum design); (ii) student individual characteristics (e.g. sex, age); (iii) student personal values (e.g. personal preferences); (iv) career needs (e.g. status, work-life balance) and (v) perception of specialty characteristics (e.g. difficulty of securing training post, or curricular activity including electives) [16]. An important insight from this research was that career preferences appear to evolve as students progress through medical school, moving from personally-based influences to more specialty-based characteristics – perhaps gleaned from clinical experience, e.g. when on student rotations [16]. Furthermore, despite a high proportion of doctors changing their specialty choice between first and final year of medical school [14], a specialty preference at the end of the degree phase is considered a good predictor of eventual long-term career [17] particularly for General Practice [24]. However, most career choice studies are cross-sectional in design and rely on respondent recall to establish the earlier career preference [16]. Such studies are vulnerable to recall bias [25], risking a distortion of memory about earlier preferences [16]. While many medical career-change studies acknowledge the impact of recall bias as a limitation of their study [26–28], to the best of our knowledge no previous study has attempted to measure the influence of recall bias on findings using a cross-sectional, longitudinal study design.

The aims of this research were to: (i) identify the factors medical graduates considered important influences in their choice of specialty after their first year of practice, and (ii) test the validity of relying on respondent recall to measure changes in specialty choice. It was hypothesised that (i) factors of influence will differ depending on specialty choice and (ii) when specialty choices are recalled and compared to previously reported choices, recall bias will become evident.

## Methods

### Study design and settings

Ireland’s health service and medical workforce configuration comprises a primary care system staffed by General Practitioners (GPs) acting as ‘gate-keepers’ to a range of secondary acute services which include hospital specialists (Medicine, Surgery, Paediatrics etc.) and diagnostic and support services. Ireland has two medical study pathways: a 5–6-year Direct Entry Medicine (DEM) undergraduate programme, mainly aimed at school leavers; and a 4-year Graduate Entry Medicine (GEM) programme for those with a degree. The latter was introduced in 2007 to make Ireland self-sufficient in respect to its medical workforce. As a result, the number of Irish and other European Union (EU) graduates of Irish medical schools more than doubled from 370 in 2006 to 854 in 2016 (with approx. one third in the GEM programme [29]). Both DEM and GEM graduates undertake an additional 1-year internship (typically comprising 6 months medicine and 6 months surgery) in an accredited Irish hospital to be awarded the ‘Certificate of Experience’ required by the Medical Council of Ireland to register as a medical practitioner in the State.

### Participants

The sampling frame was all Irish/EU final year medical students for the year 2016–17 (*n* = 725), comprised of 56% women and 66% DEM students, 95% of Irish interns are drawn from Irish/EU final year medical students [30]. As it was not feasible to pre-select and target only the Irish/EU students with priority access to internship places in Ireland [31], both Irish/EU and non-EU nationals in all six Irish medical schools were invited to complete the baseline survey (*n* = 1100).

Baseline data (baseline final med survey) were collected between November 2016 and February 2017, halfway through the final year of their medical degree using an online web-based survey provider.

The second survey (follow-up intern), the results of which are presented here, took place approximately 18 months later in June 2018, in the final month of the compulsory internship year. It was administered to the Irish/EU baseline respondents who had consented to be followed-up and provided contact details for this purpose.

Ethics approval was obtained from the Royal College of Surgeons in Ireland (reference REC1252b). Written informed consent was obtained from all respondents, in compliance with General Data Protection Regulations [32] requirements.

### Data collection

#### Outcome variables

There were two outcome variables: choice of specialty, and recall bias. For choice of specialty, both the baseline final med and follow-up intern surveys asked respondents to select their intended long-term career (first outcome variable) from a list of 14 specialty choices (see Supplementary Table A). For recall bias, the follow-up intern survey asked if the respondent’s choice of specialty had changed or remained the same since the baseline final med survey. Comparison of career choice at each timepoint, and response to the change of specialty item determined recall bias (second outcome variable).

For additional analyses, the initial 14 specialty choice options were collapsed into five categories based on those used by Ireland’s Health Service Executive’s (HSE) National Doctor Training and Planning (NDTP) unit: General Practice, Surgery, Anaesthetics, Psychiatry and Medicine (which combined options Medicine, Geriatrics, Obstetrics and Gynaecology, Paediatrics, Global International Health and Pathology). (See Supplementary Table A).

#### Predictor variables

Responses to factors of influence were measured on a four-point Likert scale: ‘not at all important’, ‘not very important’ (later collapsed to ‘not influential’), and ‘important’ and ‘very important’ (collapsed to ‘influential’).

Migration intentions following internship [2] were measured using a three-point scale: ‘Go abroad to practice medicine, but return to Ireland to continue my medical career’, ‘Go abroad to practice medicine’ and ‘Remain in Ireland to practice medicine’ and collapsed into ‘Go abroad’ and ‘Remain in Ireland’.

Feelings of emotional exhaustion (burnout) and depersonalization (callousness) experienced in the previous 12 months as an intern were captured using validated, single items [33, 34] measured on a 7-point scale (ranging from ‘Never’ to ‘Every day’) and collapsed into ‘Daily or weekly’ and ‘One time a month or less’.

Demographic information was gathered, together with additional potential predictors including current level of debt (as a result of studying medicine) and study pathway (DEM or GEM).

#### Statistical analyses

Data were analysed using StataIC, Version 15. The variables included in the multinomial logistic regression analysis were informed by previous literature and bivariate tests of association, including Fisher’s Exact Test and Pearson’s χ^2^ test, and univariable logistic regression (as appropriate). Multinomial regression analysis focused on the top three final specialty choice categories (*n* > 20 respondents) Medicine, Surgery and General Practice, as did the marginal homogeneity and McNemar’s tests used to evaluate potential recall bias. All results are reported as statistically significant at *p* < 0.05.

## Results

### Response rate

**Fig. 1:**
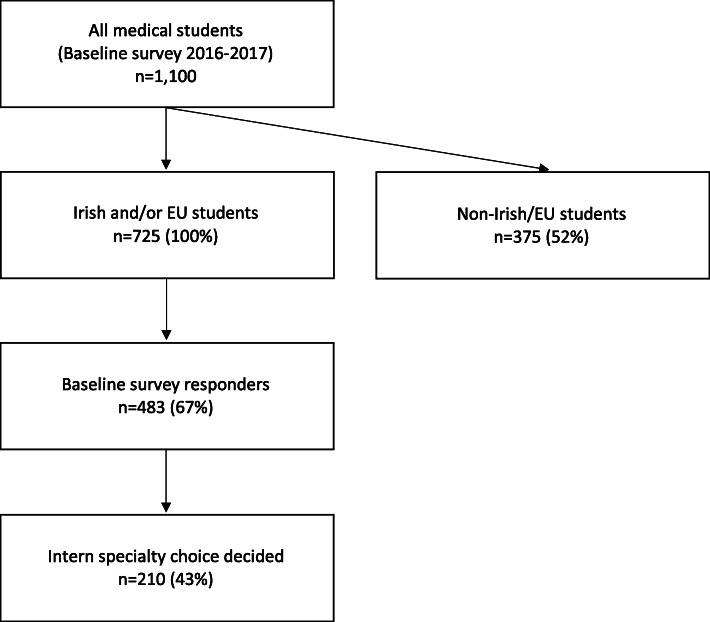
Overview of sample size

Completed surveys were returned by 67% of the sample (*n* = 483) at baseline final med. Of those, 232 (48%, or 32% of all interns) responded to the follow-up intern survey in June 2018. As the primary outcomes of interest were factors associated with specialty choice at intern, those who did not respond (*n* = 9), selected ‘not sure’ (*n* = 3) and/or ‘other’ (*n* = 10) for specialty choice at the follow-up intern survey were removed, providing a final sample size of *n* = 210 (Fig. 1) for analysis of factors of influence. One intern who selected Medicine had selected ‘Other’ for specialty choice at baseline and was therefore excluded for analysis of recall bias in change of specialty (*n* = 209).

The sample size was sufficient only to identify factors associated with the choice of the three major specialties – Medicine, Surgery and General Practice - and was not adequate to systematically examine interactions between all predictor variables.

#### Demographics

At follow-up intern, the responding interns were comprised of 54% (*n* = 113) women and *n* = 97 men, of which 65% (*n* = 130) entered study by DEM. The responding intern sample was considered representative of the overall sampling frame (56% women and 66% DEM).

The average age was 26 years (IQR 24–28). Analysis of variance showed a significant difference in age by specialty choice *F*(4, 192) = 6.58, *p* < 0.001, with Bonferroni adjusted tests showing those choosing General Practice (M = 28.6) being significantly older than those choosing Surgery (M = 25.9), *p* < 0.001; Medicine (M = 26.2), *p* = 0.001, and Anaesthesia (M = 25.3), *p* = 0.002. Analysis of variance showed GEM doctors (M = 29 years) were significantly older than DEM doctors (M = 25 years) *p* < 0.001).

### Specialty choice

The top five (of initial 14 options, see Supplementary Table A) specialty choices at each time point are presented in Table 1.

**Table 1:** Top five specialty choice at each time point: n (%)

	Baseline final med	Follow-up intern
1.	Medicine 35 (17)	Medicine 54 (26)
2.	Undecided 35 (17)	Surgery 34 (16)
3.	Surgery 31 (15)	General Practice 28 (13)
4.	General Practice 24 (11)	Anaesthesia 16 (8)
5.	Paediatrics 23 (11)	Paediatrics 14 (7)

#### Factors of influence – all specialties

**Fig. 2:**
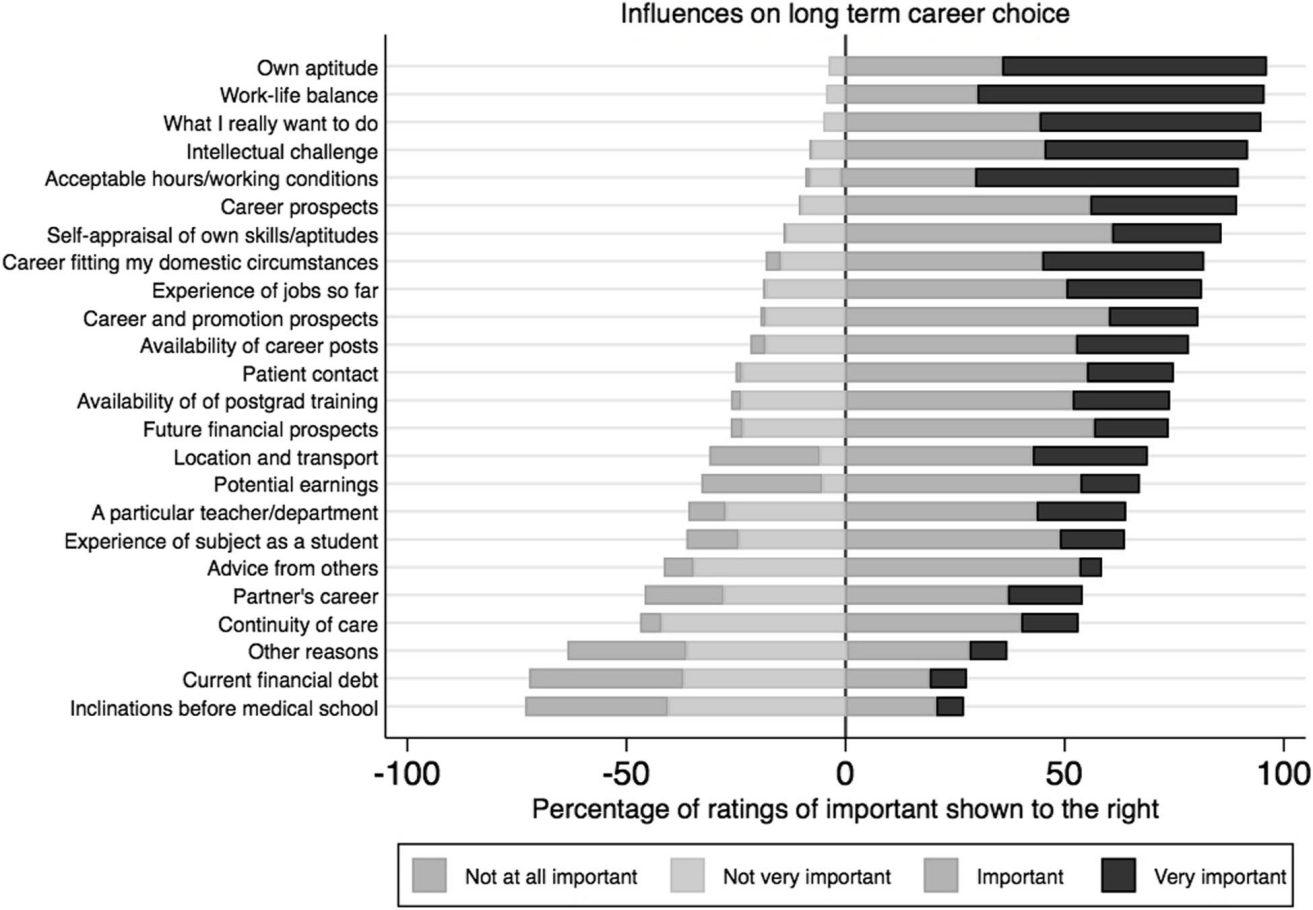
Likert plot of ratings of importance of factors of influence on long term specialty choice (*n* = 200)

Figure 2 presents the responses to the rating of factors of influence. The shaded bars to the right of the centre line represent the percentage of respondents who rated the factor ‘important’ (light) and ‘very important’ (dark). The shaded bars to the left of the centre line represent the percentage of respondents who rated the factor as ‘not very important’ (light) and ‘not at all important’ (dark).

The five factors rated as most influential to the respondents were ‘Own aptitude’, ‘Work-life balance’, ‘What I really want to do’, ‘Intellectual challenge’ and ‘Acceptable hours/working conditions’ – with over 90% of respondents rating these factors as ‘important’ or ‘very important’. The two factors rated as least important influences were ‘Current financial debt’ and ‘Inclinations before medical school’, rated by over 75% of respondents as ‘not at all important’ or ‘not very important’.

#### Change of specialty

Table 2 presents the top most popular choices of speciality made at baseline final med cross-tabulated with choice made at follow-up intern survey, 18 months later. The specialty choice of Medicine and Surgery were most consistent across the two time periods: of those who chose Medicine (*n* = 75) at baseline final med, 81% (*n* = 61) remained with Medicine as first choice in the follow-up intern survey. Similarly, of those who chose Surgery at baseline final med (*n* = 43), 86% (*n* = 37) chose Surgery 18 months later.

**Table 2:** First choice top three specialty (NDTP categories) at follow-up intern survey by first choice specialty at baseline survey

	Follow –up intern specialty choice			
	GP	Surgery	Medicine	Total
**Baseline final med specialty choice**	n (%)	n (%)	n (%)	
**GP**	*17 (71)*	3 (12)	4 (17)	24 (100)
**Surgery**	2 (5)	*37 (86)*	4 (9)	43 (100)
**Medicine**	7 (9)	7 (9)	*61 (81)*	75 (100)
**Total**	26 (18)	47 (33)	69 (49)	142 (100)

KEY: GP General Practice

Italics = same choice at both timepoints

‘Undecided’ or ‘unknown’ at either timepoint excluded

Specialty category at follow-up intern survey (*n* > 20) only

The overall balance between the three specialties remains similar at each timepoint (marginal homogeneity *p* = 0.421) with participants switching away from a specialty being balanced by others switching into it.

Additional analysis of specialty choice at each timepoint found that DEM graduates were less likely than GEM graduates to choose GP at baseline final med (*p* = 0.025).

#### Recall bias

For the analysis of recall bias those with ‘undecided’ specialty choice at baseline final med (*n* = 36) were excluded. During the follow-up intern survey, once respondents indicated their choice of specialty, they were asked if their specialty choice had changed since they had graduated (Yes/No). Twenty-eight percent (*n* = 49) responded that they had changed their specialty choice since graduation.

Table 3 presents levels of consistency between the recall (captured in the follow-up intern survey) and original first choice specialty selection made at baseline final med survey. Of the 49 respondents (28%) reporting a change of specialty, 13 (27%) of these selected the same specialty in the two surveys; of the 121 (69%) respondents reporting no change in specialty, 22 (18%) chose a different specialty at the follow-up intern survey. McNemar’s test for symmetry indicates that recall accuracy was not influenced by whether a change in specialty choice was made or not (*p* = 0.175).

**Table 3:** Analysis of recall bias in relation to choice of specialty at baseline final med and follow-up intern surveys

	(Intern) Has your specialty changed since you graduated?			
	Yes	No	Can’t remember	Total
Baseline final med specialty choice = Follow-up intern specialty choice	13 (27)^a^	99 (82)	2 (50)	114 (66)
Baseline final med specialty choice ≠ Follow-up intern specialty choice	36 (73)	22 (18)^a^	2 (50)	60 (34)
	49 (100)	121 (100)	4 (100)	174 (100)

^a^inconsistent results

Note: ‘Undecided’ or ‘unknown’ at both time points excluded

#### Factors of influence in long term specialty choice

Analyses of influences on specialty choice, as reported in the follow-up intern survey, were restricted to the three categories of specialty with *n* > 20 respondents: General Practice, Medicine and Surgery. Table 4 presents predictor variables with significance at the 95% level for associations with these specialties.

**Table 4:** Profile by specialty choice categories with *n* > 20 responders (*n* = 184)

	GP	Surgery	Medicine	Total	Chi square
	n (%)	n (%)	n (%)	n (%)	***p***
**Total Total**	28 (15)	61 (33)	95 (52)	184 (100)	
**Age** 27 years+	18 (69)	19 (34)	28 (30)	65 (37)	**0.001**
**Sex** Male	9 (35)	33 (59)	36 (39)	78 (45)	**0.030**
**Debt** €10 k or more	18 (69)	30 (54)	44 (47)	92 (52)	0.139
**Study pathway**					
Direct Entry Medicine (DEM)	11 (42)	36 (64)	66 (70)	113 (64)	**0.032**
Graduate Entry Medicine (GEM)	15 (58)	20 (36)	28 (30)	63 (36)	
**Migration intention**					
Remain in Ireland	16 (57)	26 (43)	30 (32)	72 (39)	**0.041**
Go abroad (LBR & LP)	12 (43)	35 (57)	65 (68)	112 (61)	
**“I felt burned out from my work"**					
Daily or Weekly	4 (9)	13 (30)	26 (60)	43	
Once a month or less	23 (17)	43 (32)	68 (51)	134	
**“I have become more callous toward people since I took this job”**					
Daily or weekly	8 (13)	22 (36)	31 (51)	61	0.623
Once a month or less	19 (16)	34 (29)	63 (54)	116	
**Following rated as Important/Very important**					
Own aptitude/skills	26 (15)	59 (34)	91 (52)	176	0.286
Work-life balance	27 (16)	53 (31)	91 (53)	171	0.207
What I really want to do	20 (14)	46 (32)	77 (54)	143	0.196
Intellectual satisfaction	20 (12)	55 (33)	91 (55)	166	**0.001**
Acceptable hours/working conditions	23 (17)	38 (28)	75 (55)	136	**0.006**
Career prospects	21 (13)	53 (33)	86 (54)	160	0.136
Self-appraisal of own skills/aptitude	16 (12)	42 (33)	70 (55)	128	0.088
Career fitting my domestic circumstances	23 (19)	33 (27)	66 (54)	122	**0.006**
Experience of job so far	15 (12)	42 (34)	65 (53)	122	0.075
Career and promotion prospects	13 (10)	41 (33)	70 (56)	124	**0.002**
Availability of career posts	17 (15)	36 (31)	64 (55)	117	0.793
Amount of patient contact	24 (17)	41 (29)	77 (54)	142	0.085
Availability postgraduate training places	15 (13)	37 (33)	61 (54)	113	0.511
Future financial prospects	17 (16)	37 (34)	55 (51)	109	0.583
Location and transport	15 (12)	41 (34)	66 (54)	122	0.336
Potential earnings	20 (17)	40 (33)	60 (50)	120	0.546
A particular teacher/department	7 (7)	31 (30)	64 (63)	102	**0.000**
Experience of subject as a student	14 (14)	31 (31)	54 (54)	99	0.828
Advice from others	9 (10)	26 (29)	55 (61)	90	**0.025**
Spouse/Partner’s career	18 (19)	36 (37)	42 (44)	96	0.054
Continuity of patient care	22 (21)	24 (23)	57 (55)	103	**0.001**
Current financial debt	9 (21)	17 (40)	17 (40)	43	0.107
Inclinations before medical school	9 (24)	8 (22)	20 (54)	37	0.092

KEY: *GP* General Practice, *LBR* Leave But Return, *LP* Leave Permanently

Bold values denote significance at the *p* < 0.05 level

Age, sex and study pathway (DEM/GEM) were each significantly associated with specialty choice. Sex was not collinear with either age or study pathway, but age and study pathway were collinear (*r* = 0.678, *p* < 0.001). When adjusted for age and sex, study pathway lost significance; however both age and sex remained significant predictors when corrected for the two remaining significant predictors. Therefore all predictors of specialty choice were corrected for both age and sex. Presented in Table 5 (unadjusted) and Table 6 (adjusted) are associations of demographic and training characteristics, and also factors rated by respondents as influential on their specialty choice at the follow-up intern survey (see Fig. 2).

**Table 5:** Multinomial logistic regression analysis of doctors choice of specialty (*n* = 184). Comparison group is *n* = 95 doctors choosing Medicine (UNADJUSTED)

	UNADJUSTED			
	General Practice (*n* = 28)		Surgery (*n* = 61)	
	RRR(95% CI)	*p* value	RRR(95% CI)	*p* value
**Age**	1.28 (1.11–1.47]	**0.001**	0.95 [0.82–1.09]	0.470
**Sex** Female (v. Male**)**	1.19 [0.48–2.96]	0.704	0.44 [0.22–0.87]	**0.017**
**Study pathway**				
GEM entry (v. DEM)	3.21 [1.31–7.86]	**0.011**	1.31 [0.65–2.64]	0.452
**Migration intention**				
Go abroad (LBR & LP) v. Remain	0.35 [0.14–0.82]	**0.016**	0.62 [0.31–1.21]	0.162
**Factors influencing career choice**				
Own aptitude/skills	0.64 [0.30–1.33]	0.235	1.21 [0.65–2.25]	0.537
Work-life balance	4.31 [1.26–14.69]	**0.020**	0.55 [0.32–0.95]	**0.031**
What I really want to do	0.58 [0.27–1.25]	0.167	0.83 [0.45–1.53]	0.559
Intellectual satisfaction	0.41 [0.22–0.78]	**0.007**	0.84 [0.50–1.41]	0.517
Acceptable hours/working conditions	7.05 [1.64–30.37]	**0.009**	0.58 [0.35–0.96]	**0.034**
Career prospects	0.45 [0.23–0.87]	**0.018**	1.10 [0.65–1.85]	0.727
Self-appraisal of own skills/aptitude	0.56 [0.27–1.17]	0.122	1.63 [0.90–2.95]	0.104
Career fitting my domestic circumstances	4.41 [1.82–10.72]	**0.001**	0.73 [0.46–1.16]	0.184
Experience of job so far	0.36 [0.18–0.73]	**0.005**	0.92 [0.54–1.55]	0.747
Career and promotion prospects	0.32 [0.15–0.68]	**0.003**	0.99 [0.55–1.76]	0.971
Availability of career posts	0.83 [0.45–1.51]	0.536	0.99 [0.62–1.60]	0.979
Amount of patient contact	1.48 [0.76–2.88]	0.243	0.57 [0.35–0.93]	**0.026**
Availability postgraduate training places	0.76 [0.41–1.41]	0.383	1.42 [0.86–2.33]	0.166
Future financial prospects	1.04 [0.55–1.96]	0.907	1.82 [1.07–3.08]	**0.026**
Location and transport	0.80 [0.49–1.31]	0.377	1.32 [0.78–1.66]	0.512
Potential earnings	1.41 [0.80–2.50]	0.239	1.36 [0.88–2.09]	0.166
A particular teacher/department	0.18 [0.09–0.36]	**0.000**	0.75 [0.47–1.19]	0.220
Experience of subject as a student	0.94 [0.55–1.62]	0.837	0.93 [0.62–1.41]	0.751
Advice from others	0.49 [0.26–0.96]	**0.037**	0.66 [0.39–1.11]	0.120
Spouse/Partner’s career	1.42 [0.90–2.24]	0.131	1.28 [0.91–1.80]	0.163
Continuity of patient care	3.44 [1.78–6.64]	**0.001**	0.61 [0.39–0.97]	**0.035**
Current financial debt	1.25 [0.77–2.04]	0.367	1.13 [0.77–1.66]	0.522
Inclinations before medical school	1.45 [0.86–2.43]	0.166	1.13 [0.75–1.71]	0.095

Bold values denote significance at the *p* < 0.05 level

KEY: *GP* General Practice, *LBR* Leave But Return, *LP* Leave Permanently, *RRR* Relative Risk Ratio, *CI* Confidence Interval

**Table 6:** Multinomial logistic regression analysis of doctors choice of specialty (*n* = 184). Comparison group is *n* = 95 doctors choosing Medicine (Adjusted for age and sex unless stated otherwise)

	ADJUSTED			
	General Practice (*n* = 28)		Surgery (*n* = 61)	
	RRR(95% CI)	*p* value	RRR(95% CI)	*p* value
**Age**^b^	1.27 [1.10–1.46]	**0.001**	0.93 [0.81–1.08]	0.374
**Sex**^a^ **Female (v. Male)**	1.17 [0.46–3.03]	0.738	0.44 [0.22–0.86]	**0.017**
**Study pathway**				
GEM entry (v. DEM)	1.35 [0.39–4.66]	0.640	3.13 [1.00–9.86]	0.051
**Migration intention**				
Go abroad (LBR & LP) v. Remain	0.61 [0.23–1.61]	0.319	0.54 [0.26–1.13]	0.102
**Factors influencing career choice**				
Own aptitude/skills	0.55 [0.24–1.24]	0.149	1.42 [0.75–2.70]	0.278
Work-life balance	3.41 [0.97–12.00]	0.056	0.60 [0.34–1.06]	0.076
What I really want to do	0.53 [0.23–1.23]	0.140	0.97 [0.51–1.84]	0.926
Intellectual satisfaction	0.35 [0.17–0.71]	**0.004**	0.88 [0.52–1.50]	0.637
Acceptable hours/working conditions	4.61 [1.03–20.60]	**0.045**	0.55 [0.31–0.96]	**0.035**
Career prospects	0.37 [0.17–0.78]	**0.009**	1.08 [0.63–1.84]	0.782
Self-appraisal of own skills/aptitude	0.61 [0.27–1.37]	0.230	1.82 [0.99–3.34]	0.055
Career fitting my domestic circumstances	3.19 [1.27–8.02]	**0.014**	0.76 [0.46–1.23]	0.258
Experience of job so far	0.40 [0.18–0.87]	**0.021**	0.93 [0.54–1.62]	0.808
Career and promotion prospects	0.23 [0.10–0.56]	**0.001**	0.98 [0.53–5.42]	0.961
Availability of career posts	0.75 [0.38–1.48]	0.411	0.98 [0.59–1.62]	0.943
Amount of patient contact	1.39 [0.67–2.89]	0.370	0.56 [0.33–0.95]	**0.033**
Availability postgraduate training places	0.74 [0.38–1.45]	0.385	1.47 [0.88–2.48]	0.144
Future financial prospects	0.89 [0.42–1.87]	0.760	1.68 [0.98–2.88]	0.059
Location and transport	0.71 [0.42–1.20]	0.197	1.31 [0.87–1.98]	0.196
Potential earnings	1.14 [0.60–2.17]	0.688	1.29 [0.83–2.01]	0.263
A particular teacher/department	0.18 [0.08–0.38]	**0.000**	0.80 [0.50–1.29]	0.364
Experience of subject as a student	0.93 [0.52–1.65]	0.795	0.96 [0.62–1.49]	0.857
Advice from others	0.39 [0.19–0.82]	**0.013**	0.68 [0.40–1.17]	0.163
Spouse/Partner’s career	1.34 [0.83–2.19]	0.233	1.34 [0.94–1.91]	0.111
Continuity of patient care	3.20 [1.59–6.41]	**0.001**	0.63 [0.39–1.02]	0.061
Current financial debt	0.99 [0.57–1.72]	0.964	1.22 [0.81–1.85]	0.346
Inclinations before medical school	1.13 [0.64–2.00]	0.673	1.11 [0.72–1.72]	0.640

Bold values denote significance at the *p* < 0.05 level

KEY: *GP* General Practice, *LBR* Leave But Return, *LP* Leave Permanently, *RRR* Relative Risk Ratio, *CI* Confidence Interval

^a^Adjusted for age ^b^Adjusted for sex

Compared with those who selected Medicine, those who selected General Practice were older, and those selecting Surgery were more likely to be men. Study pathway and migration intention were not associated with specialty choice.

When adjusted for age and sex, the top three factors of importance for this cohort – own aptitude, work-life balance and what I really want to do (Fig. 2) – did not differentiate between specialties. Those choosing General Practice gave significantly different ratings to those choosing Medicine: they were less likely to rate intellectual satisfaction as important; four times more likely to rate acceptable hours/working conditions as important, and less likely to rate career prospects as important. There were significant differences also for other ‘top ten’ career choice influencing factors: General Practice were three times more likely than Medicine to rate as important wanting a career that fitted their domestic circumstances; and a career that provided continuity of patient contact. General Practice did not rate as important: experience of the job so far, career and promotion prospects, the influence of a particular teacher or department or advice from others. Those choosing Surgery were not significantly different to those choosing Medicine in respect to most factors. They were, however, less likely to rate acceptable hours/working conditions and amount of patient contact as important; but were more likely to rate mentoring support as important in their choice of Surgery.

## Discussion

Seen as a ‘Generation Y’ effect [14], doctors born between 1981 and 2000 and now entering the medical workforce are viewed as having different learning styles and work-life expectations than earlier generations [35–37]. They are more self-reliant, personally oriented, socially oriented, with more focus on work-life balance than previous generations [14, 38–40]. In this study, over 90% - regardless of choice of specialty - agreed the most important factors of influence on their career decision were their own aptitude, the need for work-life balance and their personal drive (‘what I really want to do’).

However, our results present different demographic and motivational profiles by choice of specialties and are consistent with recent studies suggesting a distinct profile for doctors intending to enter General Practice in comparison to other specialties [15, 24, 41]. They tend to be older, women, GEM graduates and more likely to intend to remain in Ireland following internship. Compared with Medicine, General Practice rated as more important continuity of patient care, acceptable working hours/conditions, and having a career that fitted their domestic circumstances. In comparison, those planning to specialise in Surgery rated as less important patient contact and working hours/conditions.

Our results indicate that, in Ireland, GEM graduates are more likely to undertake General Practice and are more likely (than DEM graduates) to continue in their first choice of General Practice. Coupled with our earlier finding that GEMs are less likely to emigrate on graduation [2], this suggests that by increasing GEM places, the proportions and numbers of graduates specialising in General Practice in Ireland might increase. Other countries, including England, France and Canada, have also sought to increase the number of post-graduate training places in General Practice in response to, and to offset for, the forecasted shortage [42].

However, any increase in places cannot be made in isolation of the stressful working conditions experienced by doctors in Ireland [1, 10, 43, 44], in the United Kingdom [6] and elsewhere [5, 7]. The high levels of burnout and callousness in this cohort, which were associated with an intention to emigrate following internship [2], were distributed almost equally across the three specialty options. If these conditions are not addressed, it appears that Ireland, and other countries, will continue to lose their domestically-trained, ‘Generation Y’ doctors to the active global competition that is taking place to recruit the best and brightest of these internationally mobile medical students [2, 7, 10, 45]. The findings from this study point to the importance of regular monitoring of medical graduates’ specialty preferences during their postgraduate training years – and the need to address, where possible, the negative experiences influencing these choices.

Our findings also quantify the extent of recall bias in surveys of career choice, and confirm that recall accuracy is not influenced by whether or not a change in specialty choice has been made. This points to the necessity for the use of longitudinal studies to examine specialty career choice and change [14, 16, 46]. Medical workforce planning requires accurate estimates and robust modelling of the specialty choices of doctors coming through postgraduate training pipelines so as to maintain the supply of GPs and hospital specialists needed for an efficient health service [47, 48]. This requirement, coupled with the notoriously low response rates in workforce research, suggests the effect of recall bias must be acknowledged and addressed when designing future studies. Current literature suggests that students’ specialty preferences at the beginning of their studies are more personally focused, whereas preferences at the end of medical school are more specialty-oriented [16]. Whilst calls for further research into exactly how, and why, students change their specialty choice continue [14, 16, 17], it is widely accepted that specialty choice at the end of the degree phase is a good predictor of eventual long-term career [17]. However, many studies of the evolution of specialty choice rely on respondents’ recall to establish their baseline choice of specialty. Our findings suggest that this approach will result in recall bias. By overlooking the potential effect of recall bias, studies may be reporting skewed results and misinterpreting the dynamics of exactly how specialty choices evolve.

To facilitate reliable, cost-effective, longitudinal surveying it is necessary to implement a system that employs routine data linkage - tracking forward each annual intake of medical students through medical school and into postgraduate training period. Longitudinal study designs will not only guard against recall bias but also provide more robust medical workforce models to inform and direct recruitment drives and interventions in this area [14, 16, 49].

### Limitations

The sample size was relatively small, partly because the sampling frame for the intern survey was restricted to those Irish/EU Final Med students who had completed the baseline survey, agreed to follow-up and supplied contact details for this purpose. The introduction of the new General Data Protection Regulations (GDPR) [32] 1 month prior to the intern survey, precluded contacting interns who had not consented 18 months previously to be followed up. Consequently, generalisation of findings and statistical power were limited. However the longitudinal design has meant that recall bias could be identified and highlighted as a potential confounder in research in this area.

## Conclusions

As Ireland looks to implement a national workforce strategy for a new primary and community care-driven health system [50], an understanding of, and measures to ensure, a balanced postgraduate training pipeline of General Practitioners and hospital specialists are a priority. Results suggest that further longitudinal studies are required to identify exactly how recently qualified doctors arrive at their final choice of specialty, and what factors influence specialty choices. To prepare and address the upcoming crisis in Organisation for Economic Co-operation and Development (OECD) primary care workforce numbers, additional longitudinal research is needed to inform the recruitment of students, and to assist in the development of strategies to successfully train and retain domestically-trained medical workforces in line with national medical workforce policies.

## Supplementary Information

**Additional file 1: Table S1**. All specialty categories collapsed into HSE NDTP specialty categories.

## Data Availability

The datasets used and/or analysed during the current study are available from the corresponding author on reasonable request.

## Abbreviations

CI: Confidence Interval
DEM: Direct Entry Medicine (undergraduate programme of study)
EU: European Union
GDPR: General Data Protection Regulation
GEM: Graduate Entry Medicine (post graduate programme of study)
GP: General Practice
GPs: General Practitioners
HSE: Health Service Executive
IQR: Inter-quartile range
LBR: Leave but return (Migration intention following internship)
LP: Leave permanently (Migration intention following internship)
NDTP: National Doctor Training & Planning unit
OECD: Organisation for Economic Co-operation and Development
RRR: Relative Risk Ratio
SD: Standard deviation
WHO: World Health Organisation
