# Supplementary material for: Factors influencing specialty choice and the effect of recall bias on findings from Irish medical graduates: a cross-sectional, longitudinal study
Source: BMC Med Educ. 2020 Dec 2;20:485. doi: 10.1186/s12909-020-02405-w (PMC7709240; doi:10.1186/s12909-020-02405-w)
Supplement: Supplementary file 1 — Additional file 1: Table S1. All specialty categories collapsed into HSE NDTP specialty categories. [file 12909_2020_2405_MOESM1_ESM.docx]

**Supplementary Table A** All specialty categories collapsed into HSE NDTP specialty categories

| **Specialties categories** | |
| --- | --- |
| **Baseline final med & Follow-up intern 14 options presented** | **HSE NDTP categories** |
| General Practice | General Practice |
| Emergency Medicine  Ophthalmology  Radiology  Surgery  Paediatric Surgery | Surgery |
| Anaesthetics | Anaesthetics |
| Medicine  Geriatrics  Obstetrics and Gynaecology  Paediatrics  Global International Health  Pathology | Medicine |
| Psychiatry | Psychiatry |

KEY: HSE Health Service Executive , NDTP National Doctors Training and Planning
